# Akt-mediated phosphorylation controls the activity of the Y-box protein MSY3 in skeletal muscle

**DOI:** 10.1186/s13395-015-0043-9

**Published:** 2015-05-29

**Authors:** Luciana De Angelis, Sreeram Balasubramanian, Libera Berghella

**Affiliations:** DAHFMO, Unit of Histology and Medical Embryology, University La Sapienza, Via Scarpa 16, Rome, 00161 Italy; Division of Biology, California Institute of Technology, 1200, E. California Blvd, 156-29, Pasadena, CA 91125 USA; IRCCS Fondazione Santa Lucia, Epigenetics and Regenerative Medicine, Via Del Fosso di Fiorano, 64, Rome, 00143 Italy

**Keywords:** MSY3, Csda, Transcription factor, Akt, Myogenin, Muscle differentiation, Postnatal muscle

## Abstract

**Background:**

The Y-box protein MSY3/Csda represses myogenin transcription in skeletal muscle by binding a highly conserved cis-acting DNA element located just upstream of the myogenin minimal promoter (myogHCE). It is not known how this MSY3 activity is controlled in skeletal muscle. In this study, we provide multiple lines of evidence showing that the post-translational phosphorylation of MSY3 by Akt kinase modulates the MSY3 repression of myogenin.

**Methods:**

Skeletal muscle and myogenic C2C12 cells were used to study the effects of MSY3 phosphorylation *in vivo* and *in vitro* on its sub-cellular localization and activity, by blocking the IGF1/PI3K/Akt pathway, by Akt depletion and over-expression, and by mutating potential MSY3 phosphorylation sites.

**Results:**

We observed that, as skeletal muscle progressed from perinatal to postnatal and adult developmental stages, MSY3 protein became gradually dephosphorylated and accumulated in the nucleus. This correlated well with the reduction of phosphorylated active Akt. In C2C12 myogenic cells, blocking the IGF1/PI3K/Akt pathway using LY294002 inhibitor reduced MSY3 phosphorylation levels resulting in its accumulation in the nuclei. Knocking down Akt expression increased the amount of dephosphorylated MSY3 and reduced myogenin expression and muscle differentiation. MSY3 phosphorylation by Akt *in vitro* impaired its binding at the MyogHCE element, while blocking Akt increased MSY3 binding activity. While Akt over-expression rescued myogenin expression in MSY3 overexpressing myogenic cells, ablation of the Akt substrate, (Ser126 located in the MSY3 cold shock domain) promoted MSY3 accumulation in the nucleus and abolished this rescue. Furthermore, forced expression of Akt in adult skeletal muscle induced MSY3 phosphorylation and myogenin derepression.

**Conclusions:**

These results support the hypothesis that MSY3 phosphorylation by Akt interferes with MSY3 repression of myogenin circuit activity during muscle development. This study highlights a previously undescribed Akt-mediated signaling pathway involved in the repression of myogenin expression in myogenic cells and in mature muscle. Given the significance of myogenin regulation in adult muscle, the Akt/MSY3/myogenin regulatory circuit is a potential therapeutic target to counteract muscle degenerative disease.

**Electronic supplementary material:**

The online version of this article (doi:10.1186/s13395-015-0043-9) contains supplementary material, which is available to authorized users.

## Background

Basic helix-loop-helix (bHLH) myogenic regulatory factors coordinate the correct function and organization of many skeletal muscle functions. These require precise control both at the transcriptional and post-transcriptional level. Among the myogenic bHLH factors, myogenin is crucial in the embryo, in fetal and in adult life, due to its many functions in regulating skeletal muscle growth, maturation and metabolism [1–10]. Although it has been reported that multiple mechanisms control myogenin in postnatal muscle [11–15], the signaling that specifically controls myogenin expression levels in adult skeletal muscle is not yet fully known. We have previously shown that MSY3/Csda binds a highly conserved cis-acting element located upstream of the myogenin promoter (myogHCE) and regulates the postnatal down-regulation of myogenin [16].

MSY3 (MSY4, YB-2, CSDA, dbpA, ZONAB) is a member of the cold shock domain (CSD) family of proteins also known as Y-box proteins, which are evolutionarily conserved proteins that function as transcription factors and regulators of RNA metabolism and protein synthesis. Y-box proteins act via diverse mechanisms, as they work by DNA binding and non-DNA binding mechanisms. Moreover, they bind single-stranded DNA and RNA and are involved in both gene repression and activation [17–20]. MSY3 is a DNA binding protein, which acts as a transcriptional repressor. It has been extensively studied in tissues where it is highly expressed, including skeletal muscle where two isoforms of MSY3 (long and short), which differ by a single exon, are present [16, 21–23]. Possible MSY3 mechanisms of action have been studied in cell culture studies. In particular, in fibroblasts MSY3 represses VEGF expression by preferentially binding single-stranded sense DNA of the hypoxia responsive region (HRR) of the VEGF promoter [24]; it regulates proliferation and density in epithelial cells, by shuttling between the tight junction and the nucleus, where it regulates the expression of cell cycle progression genes [25–27]. It is also upregulated in epithelial cancers and promotes proliferation in neoplastic cells [28, 29]. Although it is highly expressed in skeletal muscle and heart, very little is known about the role of MSY3 in the muscle gene regulatory network. In skeletal muscle we showed that MSY3 binds, *in vitro* and *in vivo*, the myogHCE sequence, a conserved regulatory module located upstream of the myogenin promoter and specifically regulates myogenin expression [16]. Additionally, myogHCE deletion analyzed using reporter transgenic mice, led to a persistence of myogenin expression along the muscle fiber during the period of muscle maturation [16]. These results show that both MSY3 and the myogHCE play a crucial role in myogenin down-regulation that occurs during the refinement of synaptogenesis, when AChR expression becomes restricted at synaptical sites in the muscle fiber [30, 31].

Akt (or protein kinase B (PKB)) is a family of versatile serine/threonine kinases (Akt1, Akt2, and Akt3). They play a pivotal role in numerous biological processes, such as cell growth, proliferation and survival, and human diseases such as cancer and type 2 diabetes. Upon its activation, which involves tyrosine kinase receptors and signaling events, Akt binds and phosphorylates a wide range of transcription factors altering their sub-cellular localization and thus leading to their activation or inhibition [32–34]. Multiple findings indicate that Y-box protein signaling involves the phosphatidylinositol 3-kinase (PI3K)/Akt pathway, which modulates their activity mainly by regulating their translocation between nucleus and cytoplasm [24, 29, 35–38]. PI3K signaling pathway is essential for skeletal muscle growth during development and regeneration after injury regulating myoblast proliferation and fusion, muscle gene expression, and post-mitotic growth of myotubes [39, 40]. In adult muscle, the Akt signaling cascade controls loss of muscle mass by inhibiting protein degradation through phosphorylation and inactivation of the FoxO family of transcription factors [41, 42]. Akt is also activated during adaptive and IGF-induced myotube hypertrophy and can prevent loss of muscle mass by promoting protein synthesis through the activation of the mTOR pathway [43–45]. Numerous direct and indirect substrates of the PI3K pathway have been characterized in skeletal muscle: key regulatory proteins involved in translation and protein synthesis, effectors of protein degradation such as ubiquitin ligases and also transcription factors and chromatin remodeling complexes involved in skeletal muscle differentiation and growth [39, 40, 46–55].

During muscle maturation, MSY3 becomes dephosphorylated and accumulates in the nuclei of muscle fibers and represses myogenin [16]. This correlates with myogenin down-regulation observed as muscle proceeds through advanced stages of postnatal life. Given the role of MSY3 in skeletal muscle differentiation and Akt’s capacity to regulate Y-box protein activity, we asked if the PI3K pathway controls MSY3 activity to repress myogenin during muscle differentiation and in adult life. In this study, by using specific inhibitors of the PI3K pathway, we showed that Akt phosphorylates the MSY3 CSD in myogenic cells and subsequently MSY3 is sequestered in the cytoplasm. Phosphorylation by Akt and subsequent cytoplasmic translocation of MSY3 results in derepression of myogenin, which is required for progression of differentiation. Akt over-expression in adult muscle induces MSY3 phosphorylation and upregulates myogenin expression. These findings in the C2C12 cell culture system and postnatal and mature muscle correlate with myogenin down-regulation observed as muscle proceeds throughout advanced stages of postnatal life. Furthermore, they suggest a role for Akt in modulating, through MSY3 phosphorylation, myogenin activity in adult skeletal muscle function.

## Methods

### Cell, treatments, transfections, and RNA interference

C2C12 cells (ATCC) were cultured in DMEM supplemented with 20 % FBS (growth medium (GM)) and induced to differentiate with DMEM supplemented with 2 % HS (differentiation medium (DM)). SB203580, LY294002 and Rapamycin were purchased from Calbiochem. Wortmannin, PD98059, KN-62 and KN-93 were purchased from Sigma. Transfections of C2C12 cells were performed with FuGene reagent (Roche). To generate and maintain stable clones, selection of C2C12 cells was performed with G418 at a concentration of 400 μg/ml and puromycin at 2 μg/ml. Gene expression was determined by qRT-PCR as described previously [16]. Down-regulation of Akt1 and Akt2 by RNA interference was achieved as previously described [55].

### Immunofluorescence

C2C12 cells were fixed in 4 % paraformaldehyde (10 min at 4 °C) and then treated with 0.15 % Triton X-100 in PBS for 5′ at RT. After re-hydration with PBS, they were blocked with 10 % NGS for 1 h at RT and then processed for immunofluorescence (IF) with an MSY3 rabbit antiserum ZONAB (Zymed) [56], diluted 1:100 in 1.5 % normal goat serum in PBS followed by Alexa Fluor 594 goat anti-rabbit (Invitrogen). For FLAG IF, a mouse anti-FLAG M2 (Sigma) was used at the dilution of 1:400, followed by Alexa Fluor 488 goat anti-mouse (Invitrogen) incubation. Also mouse anti-MHC (MF20) (Developmental Studies Hybridoma Bank) and rabbit anti-histoneH3 (Sigma) were used. IF images were exported in Tiff format, and fluorescence intensities in the nuclear and cytoplasmic regions were quantified using ImageJ. Background-corrected N/C ratios were calculated from mean fluorescence intensities measured within a small square or circular region of interest placed within the nucleus, cytoplasm, and outside of each cell. Due to LY294002 effects on proliferation, measurements on LY 80 % (Fig 3) were performed only in those areas where the cells reached confluence. Cross sections of limbs of 2-day-aged pups (2 pn) and TA of 1-month-aged mice (1 M) were fixed in methanol for 10 min at −20 °C and then treated with 0.2 % Triton X-100 in PBS for 5′ at RT. After re-hydration with PBS, they were blocked with 4 % BSA for 1 h at RT and then processed for immunofluorescence (IF) with ZONAB, diluted 1:100, and mouse anti-caveolin 3 (BD), diluted 1:500, in 4 % BSA-PBS followed by Alexa Fluor 594 goat anti-rabbit and Alexa Fluor 488 (Invitrogen).

### Immunoblot analysis and immunoprecipitation

Nuclear and cytosolic protein extracts from C2C12 cells and from skeletal muscle were isolated with the Nuclear Extract Kit (Active Motif). Proteins were fractionated by electrophoresis on 10 % Novex Tris-glycine polyacrylamide gel (Life Technologies). For MSY3 phosphorylation analysis, 8 % Novex Tris-glycine polyacrylamide gels were used (Life Technologies). They were electroblotted to nitrocellulose membrane and reacted with rabbit anti-MSY3 (ZONAB) (Life Technologies), mouse anti-FLAG M2 (Sigma), mouse anti-α − tubulin and mouse anti-GAPDH (Sigma-Aldrich), mouse anti-myogenin (FD5, IOWA Hybridoma bank), anti-phospho-Akt (Ser473), and anti-Akt (Cell Signaling). Immunoreactivity was determined using the ECL method (Life Technologies) with the reagents provided according to the manufacturer’s instructions. Densitometry calculations were done by measurements of band intensities using ImageJ, verifying for background and non-saturation. Values are expressed as the integrals (area*means density) of each band (normalized to weakest band).

For immunoprecipitation (IP), protein extracts (50 μg) from skeletal muscle (8 and 15 pn) isolated as described above, were incubated for 2 h at 4 °C with 2.5 μg of anti-MSY3 Ab, ZONAB, previously coated to Dynabeads® M-280 Sheep anti-Rabbit IgG (Life Technologies). The immunocomplexes were analyzed by Western blot as described above, together with not immunoprecipitated extract (Input) and unconjugated IgG.

### Protein dephosphorylation assay

Forty micrograms of extracts were incubated with different amounts of Antarctic Phosphatase (Roche), in the presence of its reaction buffer 10× for 30 min at 37 °C and directly analyzed or immunoprecipitated and analyzed by Western blot as described above.

### Plasmids

The MSY3FLAG and mutated forms were generated by PCR amplification from the parental pcDNA3-MSY3 HA plasmid (16) and then cloned in frame in the N-p3XFLAG-CMV vector using the following oligos: FLAGFOR 5′GAATTCGAGCGAGGCGGGCGAGGCCACC 3′; FLAGREV 5′TCTAGACGGTGCCTGGGAGCCAGGGTC 3′. GST-MSY3 fusion proteins were generated as previously described [16].

### Preparation of recombinant and nuclear protein and gel shift analysis

EMSA experiments were performed according to protocols previously described [16]. Reactions were analyzed on 8 % non-denaturing polyacrylamide gel.

### Analysis of phosphorylated recombinant CSD protein

The *Escherichia coli* strain BL21 transformed with pGST-MSY3 was induced with isopropyl-thio-β-D-galactopyranoside to produce recombinant GSTMSY3 protein. The fusion proteins were purified on glutathione-sepharose beads as described by the manufacturer (Promega). Phosphorylation of MSY3 was determined by incubating 50 ng of recombinant GSTMSY3 protein with Akt and GSK3β active proteins (Upstate), in a mixture containing 10 mM MgCl2, 2 mM DTT, 5 mM β-glycerolphosphate, 0.1 mM Na3VO4, 25 mM Tris–HCl, pH 7.5 and 50 μCi [γ ^32^P] ATP in an *in vitro* phosphorylation assay. Kinase reactions were incubated at 30 °C for 30 min, and ^32^P labeled proteins were examined by 12 % SDS/PAGE and detected after exposure of gels to X-ray films. For preparation of cold kinase proteins, kinase reactions were performed by incubation with or without active Akt and GSK3β, in presence of 0.5 mM ATP.

### Site-directed mutagenesis

The FLAGMSY3 mutant constructs Ser126Ala and Ser328Ala were synthesized and sequenced by GenScript Corporation (Piscataway, NJ, USA; www.genscript.com).

### Chromatin immunoprecipitation assay

ChIP in C2C12 cells was performed as previously described [16], and in adult muscle, the following modifications were introduced: 100 mg of TA from an adult mouse (3-month-aged 3MM) was dissected 7 days post-electroporation, minced, and then fixed with 1 % formaldehyde for 15 min. After fixation, glycine was added to a final concentration of 0.125 M. The tissue was then homogenized in PBS using the Micro-Dismembrator U (Sartorius). Nuclei were collected in lysis buffer and sonicated. The average size of the fragments was approximately 400 bp. We used 2 μg of anti-ZONAB/MSY3 (Life Technologies) and qRT-PCR was performed as described above. Primers used for amplification were as follows: myogenin promoter Forward 5′-CCCTGCCCCACAGGGGCTGTG-3′ Reverse 5′-ACGCCACAGAAACCTGAGCCC-3′; IGH enhancer Forward 5’-GCCGATCAGAACCAGAACACCTGC-3′Reverse 5′-TGGTGGGGCTGGACAGAGTGTTTC-3′.

### *In vivo* electroporation

Animal experiments were conducted after approval from the Institutional Animal Use and Care Committee (IACUC) at California Institute of Technology, (protocol n.1565). TA muscle was electroporated as previously described [16]. Expression vectors were injected with a 0.5-m insulin syringe through a 27-gauge needle into the TA muscles of 3MM C57BL6 mice (20 μg of myristoylated Akt expressing vector or pcDNA3 in a constant volume of 20 μl of PBS).

## Results

### MSY3 is progressively dephosphorylated as skeletal muscle matures

**Fig. 1:**
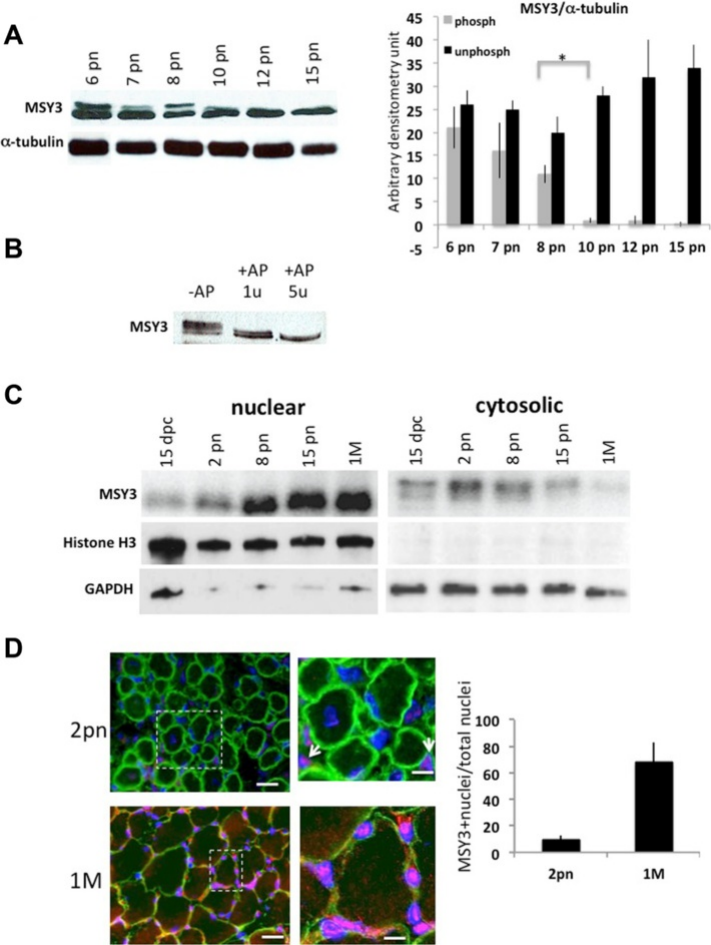
MSY3 is progressively dephosphorylated and accumulates in the nuclei in skeletal muscle after birth. **a** Left: Western blot with anti-MSY3 (ZONAB) protein extracts of limb and TA muscle at different postnatal days (pn). α-tubulin was used as normalizer. Right: densitometry calculations for MSY3 faster (dephosph) and slower (phosph) migration bands of the Western blot on the left, normalized by α-tubulin. Figure displays results representative from three independent experiments. *P <0.01 by Student’s *t* test. **b** Western blot with extracts from 15 dpc embryo limbs treated with Antarctic Phosphatase (AP), 1 unit and 5 units, and probed with anti-MSY3 Ab (ZONAB). **c** Western blots of nuclear and cytosolic fractions of protein extracts of limb muscle at fetal (15 dpc), postnatal (pn), and mature, 1 month (1 M), stages. Histone H3 was used as normalizer of the nucleic fraction and GAPDH as normalizer of the cytosolic fraction. **d** Left: expression of MSY3 evaluated by IF with anti-MSY3 Ab, ZONAB, in cross sections of limbs of 2-day-aged pups (2 pn) and TA of 1-month-aged mice (1 M). The images merge MSY3 expression (red), caveolin 3 expression (green), and Hoechst nuclei staining (blue). MSY3 localization in nuclei is labeled in purple. Scale bars = 100 μm. On the right, high magnification images (corresponding to *dotted white squares*) show staining of few muscle fibers. Scale bar = 40 μm. *White arrows* indicate sporadic MSY3 nuclear accumulation in 2 pn limb cross sections. Right Graph quantifies MSY3 positive nuclei over the total nuclei in cross sections of limbs of 2-day-aged pups (2 pn) and TA of 1-month-aged mice (1 M)

We previously demonstrated that MSY3 protein expression progressively increased as muscle matures, from late stages of fetal development (15 dpc) to the mature stages of muscle growth in postnatal life (2 months old, 2MM). A Western blot analysis showed that the migration pattern of MSY3 protein (long isoform) shifts from a multi-band to a single lower molecular weight band in the nuclear extract of leg muscle, between very early stages of postnatal development (8 pn) and later stages (15 pn) [16]. We interpreted this modification as a result of the protein phosphorylation state change. We analyzed the MSY3 protein-banding pattern from 6–15 days postnatal extracts and inferred the time of shift from a multi-band to a single band migration pattern around the ninth day of postnatal life (Fig. 1a). During progression from fetal to adult stages, myogenin expression is severely reduced along the muscle fiber, as described previously [30, 57], and shown in Additional file 1: Figure S1. To investigate whether this change in MSY3 migration pattern is caused by the loss of a phosphate group, we incubated protein extracts from limbs of 16 dpc embryos with Antarctic Phosphatase (AP). Upon treatment with AP, MSY3 protein migrated as a single band compared to the multiple bands observed in the untreated sample (Fig. 1b), suggesting that MSY3 is phosphorylated during muscle development. Analysis of MSY3 immunoprecipitated protein from skeletal muscle extracts of 8 pn and 15 pn mice showed the same migration observed in skeletal muscle (Additional file 1: Figure S2A). Treatment of the immunoprecipitated MSY3 protein with AP affected its migration pattern similar to the input control (non-immunoprecipitated) MSY3 protein (Additional file 1: Figure S2B).

We then asked if the phosphorylated and dephosphorylated forms of MSY3 protein segregated in different compartments of muscle fibers throughout perinatal and mature developmental stages. Translocation from nucleus to the cytoplasm and consequent activation is indeed the main effect of phosphorylation on many transcription factors including Y-box proteins [29, 36, 58]. By nuclei-cytosol fractionation, we observed that the dephosphorylated MSY3 protein progressively accumulates in the nuclei of muscle fibers from fetal and perinatal stages to mature developmental stages (1 month old, 1 M). During the same time, we observed a corresponding reduction in the phosphorylated form of MSY3 in the cytosol (Fig. 1c). Using IF, we observed nuclear MSY3 protein accumulation in TA cross sections of younger (2 pn) and adult mice (1 month old, 1 M). The images show much more MSY3 localization in the adult muscle fibers as compared to the nuclei of muscle fibers of younger mice (Fig. 1d; Additional file 1: Figure S3). An estimation of the MSY3 positive-stained nuclei in both stages revealed 68 % of total nuclei in adult fibers as MSY3 positive while only a few interstitial nuclei (10 % of total) in the younger fibers were positive for MSY3 (Fig. 1d; Additional file 1: Figure S3). However, due to technical difficulties, we could not substantiate expression in the cytosolic fraction of the fibers as shown by the nuclei-cytosol fractionation shown in Fig. 1c. In adult muscle fiber, MSY3 expression is detectable in almost all the myonuclei and other sub-laminal and interstitial cells (Fig. 1d). Our results indicate that dephosphorylated MSY3 migrates progressively in the nucleus as the muscle grows and matures. MSY3 dephosphorylation and segregation in the nuclei occurs concomitantly with myogenin down-regulation. In retrospect, these data support the hypothesis that phosphorylation and subsequent localization of MSY3 in the cytoplasm is responsible for modulating MSY3 activity as a repressor of myogenin expression.

### MSY3 is phosphorylated by Akt in C2C12 cells

**Fig. 2:**
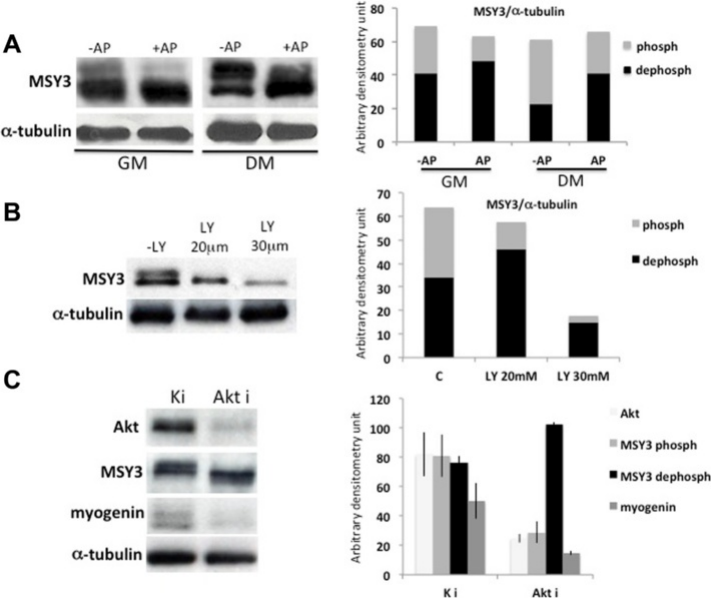
MSY3 is phosphorylated by Akt in C2C12. **a** Left: multi-band migration pattern is shown in the Western blot with protein extracts of C2C12 myoblasts (GM) and myotubes (DM) treated with 1 unit of Antarctic Phosphatase (AP), probed with anti-MSY3 Ab (ZONAB). α-tubulin was used as normalizer. Right: densitometry calculations for faster (dephosph) and slower (phosph) migration bands of the Western blot on the left, normalized by α-tubulin. **b** Left: Western blot probed with anti-MSY3 Ab (ZONAB) and anti-myogenin Ab with protein extracts of C2C12 myoblasts (GM) treated with two different (20 μM and 30 μM) concentrations of LY294002 (LY). α-tubulin was used as normalizer. Right: densitometry calculations for faster (dephosph) and slower (phosph) migration bands of the Western blot on the left, normalized by α-tubulin. **c** Left: immunoblot with an anti-Akt total, an anti-MSY3 and anti-myogenin Abs in protein extracts of C2C12 grown in DM for 36 h, transfected with scrambled RNAi oligos (Ki) and RNA interfering oligos against of Akt 1 and Akt 2 (Akti), demonstrates that Akt activity is responsible for MSY3 phosphorylation. Right: densitometry calculations for Akt, myogenin, and MSY3 faster (dephosph) and slower (phosph) migration bands of the Western blot on the left, normalized by α-tubulin. We estimated a reduction of Akt of 75 %. Figure displays results representative from three independent RNAi experiments. Akt *P* <0.01; MSY3 dephosph *P* <0.05; MSY3 phosph *P* <0.01; myogenin *P* <0.05 by Student’s *t* test

In order to determine the mechanism responsible for MSY3 phosphorylation in skeletal muscle, we analyzed MSY3 protein expression and migration patterns in C2C12 myogenic cells by Western blot analysis. MSY3 protein (long isoform), detected with the polyclonal antibody ZONAB, is expressed at the same levels in proliferating (GM) and differentiated for 48 h (DM) C2C12 cells (Fig. 2a). We observed that MSY3 migrates as multiple forms in both conditions, GM and DM. Also, densitometry measurements of untreated extracts show that the dephosphorylated form of MSY3 is more prominent in myoblasts (GM) than in myotubes (DM) (Fig. 2a). After *in vitro* treatment of C2C12 extracts with AP, MSY3 protein shifted to a single faster migrating form. This indicates that both phosphorylated and the dephosphorylated forms of MSY3 are present in C2C12 myogenic cells (Fig. 2a). We then tested the effects of LY294002 (LY), an inhibitor of the PI3K/Akt pathway that also inhibits myogenin expression and myogenic differentiation [52, 55], on MSY3 phosphorylation *in vivo* (Additional file 1: Figure S4A, B). Treatment with LY reduced MSY3 basal phosphorylation in C2C12 myoblasts (Fig. 2b). However, we observed that the highest concentration of LY tested (30 μM) resulted in a pronounced reduction of MSY3 expression. We observed no changes in the MSY3 protein phosphorylated/dephosphorylated ratio when C2C12 cells were treated with other kinase inhibitors, involved in muscle differentiation or hypertrophy such as p38 mitogen-activated protein kinase (p38MAPK) inhibitor (SB203580), mitogen-activated protein kinase kinase (MEK) inhibitor (PD98059), or calcium/calmodulin-dependent protein kinase (CAMKII) inhibitors (KN-62; KN-93) (Additional file 1: Figure S4A, B, D, E). Inhibition of the IGF1/PI3K/Akt pathway upstream of Akt by treatment with the PI3K pathway inhibitor, wortmannin, showed a reduction in MSY3 phosphorylation (Additional file 1: Figure S4C). In addition, treatment with rapamycin, the mTOR inhibitor that blocks the PI3K pathways downstream of Akt, did not alter the ratio of phosphorylated/dephosphorylated MSY3 protein (Additional file 1: Figure S4C), although both wortmannin and rapamycin inhibit myogenic differentiation (Additional file 1: Figure S4A). The observation that only the PI3K inhibitor altered the phosphorylated/dephosphorylated MSY3 levels argues for a role for Akt in phosphorylating the Y-box protein MSY3 in myogenic cells. This conclusion is in agreement with the previously documented role of Akt in phosphorylating and regulating functions of Y-box proteins in other cell types [29, 35, 38].

To demonstrate that Akt is responsible for MSY3 phosphorylation, we knocked down Akt1 and Akt2 expression by RNA interference in C2C12 cells, since both kinases are involved in regulating the myogenic program [54, 59, 60]. Upon reduction of Akt expression, estimated at 75 %, we observed a strong decrease in the amount of the phosphorylated MSY3 protein (Fig. 2c). We also observed a concomitant reduction of myogenin protein expression (Fig. 2c) and an impairment of myogenic differentiation (Additional file 1: Figure S4F). Based on these data, we hypothesize that Akt is responsible for MSY3 phosphorylation in myogenic cells and through MSY3 plays an indirect role in regulating myogenin activity.

### Dephosphorylated MSY3 accumulates in C2C12 nuclei

**Fig. 3:**
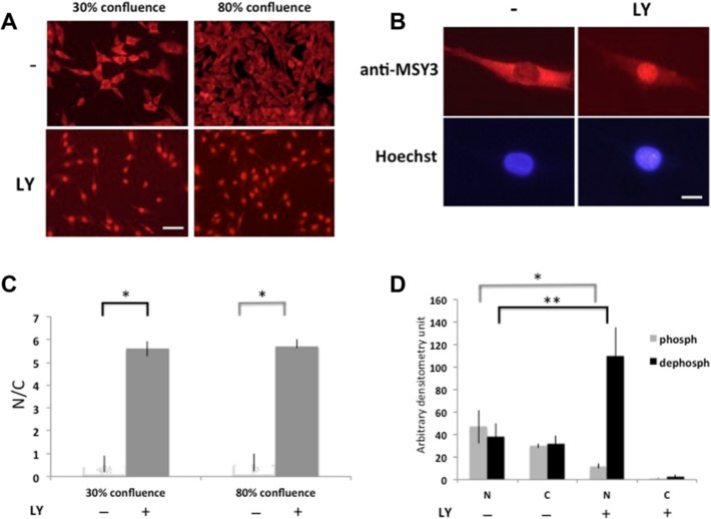
Dephosphorylated MSY3 accumulates in C2C12 nuclei. **a** Expression of MSY3 evaluated by IF with anti-MSY3 Ab, ZONAB, in C2C12 myoblasts in proliferating (30 % confluence) (IF left) and confluent (80 % confluence) growth (IF right) upon LY294002 (LY) treatment. Scale bar = 200 μm. **b** High magnification image (60×) of MSY3 expression (top) and Hoechst nuclei staining (bottom) in C2C12 myoblasts untreated (−) and upon LY treatment (LY). Scale bar = 60 μm. **c** N/C ratios of MSY3 signal in C2C12 myoblasts untreated (−) and upon LY treatment (+), evaluated as a mean ± DS for 60–80 cells from a total of five independent experiments. Quantification was performed as described in the “Methods” section. *P <0.05 by Student’s *t* test. **d** Densitometry calculations for MSY3 faster (dephosph) and slower (phosph) migration bands in nuclear (N) and cytosolic (C) compartments in untreated (−) and upon LY treatment (+), analyzed on Western blots performed as that shown in Fig S2B. Figure displays results representative from three independent RNAi experiments. *P <0.01; **P <0.001 by Student’s *t* test

Although MSY3 is a nucleic acid binding protein, in epithelial cells, it is sequestered in the cytosol by a tight junction-associated protein, ZO-1, which controls its accumulation and activity as a transcription factor in the nuclei [25]. This suggests that sub-cellular localization of MSY3 is a possible mechanism for regulating MSY3 functions. Phosphorylation by Akt is a requirement for initiating sub-cellular trafficking of many Akt targets, including the Y-box proteins [29, 36]. To investigate the role of Akt in MSY3 intracellular distribution in myogenic cells, we treated proliferating (GM) and differentiated C2C12 cells (DM) with the Akt inhibitor LY and detected MSY3 protein localization by IF with ZONAB. Fluorescence images of untreated C2C12 cells showed that MSY3 is not localized in the nuclei and is dispersed in the cytoplasm, both in myoblasts and myotubes, with some exceptions of selective nuclear localization (0.1 %, white arrow in Additional file 1: Figure S5A). To examine MSY3 localization in more detail, we quantified the extent of fluorescence observed in the cytoplasm and nucleus from confocal sections and calculated the nuclear/cytoplasmic (N/C) ratio for 60–80 cells across five independent experiments (Fig. 3c). This analysis showed that, although a high variability exists in the N/C ratio across different experiments in untreated cells, MSY3 preferentially localized in the cytosol or was equivalently distributed between nucleus and cytosol. Additionally, MSY3 localization is independent from cell confluence, since similar N/C ratios were observed in proliferating cells at 30 and 80 % of confluency (Fig. 3c). After LY treatment, we observed a robust MSY3 accumulation in the nucleus both in proliferating and differentiated cells (Fig. 3a, c and Additional file 1: Figure S5A). A higher magnification (60×) of the IF shows that in a low percentage of the nuclei MSY3 protein is completely depleted (Fig. 3b). When C2C12 cells were treated with LY in DM, myogenesis was inhibited and mono-nucleated cells with MSY3 localized in the nuclei were observed. Although, in some cases, we observed small myotubes with diffuse MSY3 staining (in nucleus and cytoplasm) (green arrow in Additional file 1: Figure S5A). We analyzed the distribution of phosphorylated and dephosphorylated MSY3 in the nuclear (N) and (C) cytoplasmic compartments in untreated and LY treated C2C12 myoblasts by Western blot. In concordance with the IF observations, densitometry measurements showed that phosphorylated and dephosphorylated forms of MSY3 protein were distributed equally between the nuclei and cytoplasm in untreated myoblasts and upon LY treatment the nuclei were enriched for the dephosphorylated form (Fig. 3d and Additional file 1: Figure S5B). These results demonstrate that Akt phosphorylation inhibited by PI3K/Akt pharmacological blockade induces a robust accumulation of dephosphorylated MSY3 in the nuclei of myogenic cells. In the nucleus, dephosphorylated MSY3 acts as a transcriptional repressor of differentiation promoting genes, such as myogenin.

### Akt phosphorylates MSY3 and reduces its binding to myogHCE *in vitro*

**Fig. 4:**
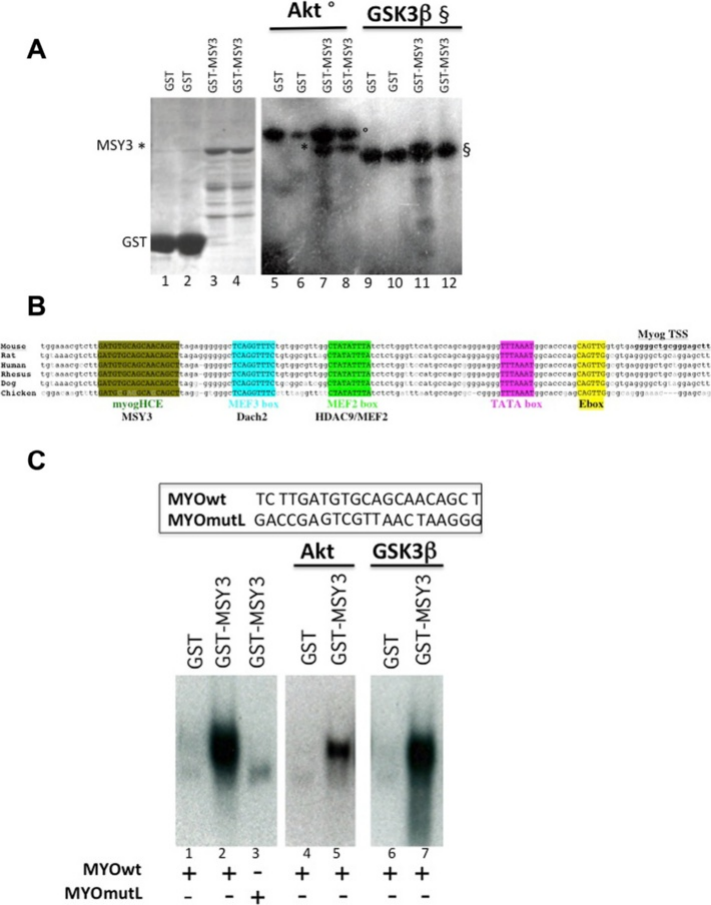
Akt phosphorylates MSY3 and reduces its binding to myogHCE *in vitro*. **a** Phosphorylation of MSY3 by Akt. GST-MSY3 (*) was phosphorylated by Akt, (lanes 7 and 8) but not by GSK3β (11 12). Auto-phosphorylated Akt protein (°) and GSK3β (§) are present. The Comassie gel is shown (lanes 1–4). For each sample, there are two experimental replicates. **b** Schematic of the myogenic locus. This diagram describes the myogenin promoter (the 130 bp upstream region from the TSS) and the genomic alignments of six vertebrates. The schematic also shows the relative locations of myogHCE element—the region to which MSY3 binds and other well-known motifs (myogHCE-brown, MEF3box-light blue, MEF2box-green, TATA box-purple, and E-box-yellow. MyogHCE is MSY3 binding site [16]; MEF3box is Dach2 binding site [13]; MEF2box is MEF2/HDAC9 binding site [12]. **c** MSY3 phosphorylation by Akt impairs its binding at the myogenin promoter. Sequences of MyogHCE (MYOwt) and mutant myogHCE (MYOmutL) oligonucleotides used in EMSA are shown. Mutant MYOmutL disrupts the myogHCE sequence, which incudes the MSY3 site. Mobility shift assay (EMSA) of GST-MSY3 binding to EMSA assay of GST and GSTMSY3 fusion protein with myogHCE (MYOwt) and mutant myogHCE (MYOmutL) ^32^P labeled oligonucleotide in double-strand form without (first three lanes) and with prior treatment by Akt (lanes 4 and 5) and by GSK3β (lanes 6 and 7)

To determine if MSY3 is a downstream phospho-substrate for Akt *in vitro*, we constructed a GST-MSY3 fusion construct with GST at the N-terminal end and tested the ability of Akt and GSK3β, another kinase regulated by the PI3K pathway [47], to phosphorylate MSY3, in an *in vitro* kinase assay (Fig. 4a). We observed that a phospho protein with the same molecular weight as GST-MSY3 (Comassie staining lanes 3–4) is present only when the GST-MSY3 protein (but not GST alone) is incubated with Akt (Fig. 4a lanes 7–8) but not when it is incubated with GSK3β (Fig. 4a, lanes 11–12). This evidence confirms that MSY3 is specifically phosphorylated by Akt in myogenic cells. MSY3 represses myogenin by binding the highly conserved DNA cis-acting element located upstream of the myogenin promoter (myogHCE) (Fig. 4b) [16]. To analyze the functional impact of MSY3 phosphorylation, we tested if the binding of MSY3 to the myogHCE regulative element *in vitro* is modulated by the Akt phosphorylation of MSY3. The GST-MSY3 protein binds efficiently to the WT myogHCE oligo, (MYOwt) but to a lesser extent to the mutated oligo (MYOmutL) as expected [16] in a gel shift assay (Fig. 4c, lanes 2–3). We observed that when MSY3 is phosphorylated *in vitro* by Akt, its binding at the myogHCE (MYOwt) element is reduced more than 50 %, while phosphorylation by GSK3β showed no effects on binding (Fig. 4c, lanes 4–7). This result indicates that MSY3 phosphorylation by Akt shows a moderate but specific effect on the DNA binding property of the MSY3 recombinant protein.

### Akt phosphorylates MSY3 in C2C12 cells, decreasing its binding activity and increasing myogenin expression

**Fig. 5:**
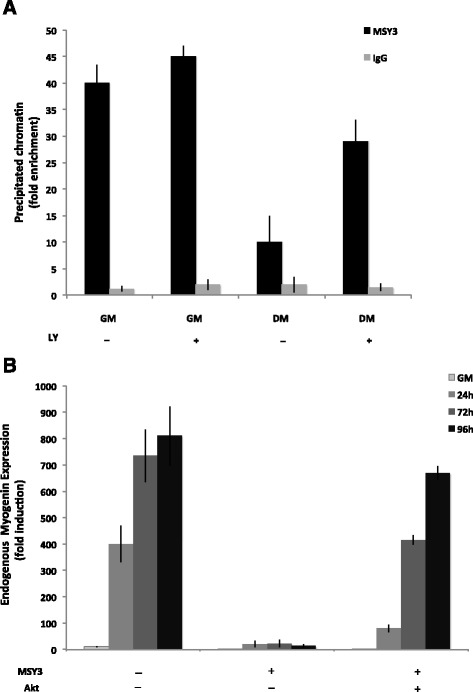
Akt phosphorylates MSY3 in C2C12 cells, decreasing its binding activity and increasing myogenin expression. **a** Chromatin immunoprecipitation (ChIP) analysis for control IgG and MSY3 antibodies used individually to enrich fixed chromatin from undifferentiated (GM) or differentiated (DM) C2C12 cells, treated (+) or not (−) with LY2940002 (LY). qRT-PCR is used to quantify sequences from the myogenin promoter. **b** Measurements of endogenous myogenin expression by qRT-PCR in mock transfected C2C12 (−MSY3, −Akt) and MSY3 over-expressing C2C12 multiclones transfected (+MSY3, +Akt) or not (+MSY3, −Akt) with myristoylated Akt in proliferation medium (GM) or upon different times cultured in differentiation medium (24–72–96 h DM). GAPDH was used to normalize expression levels

In order to further assess if Akt phosphorylation of MSY3 interferes with its binding of myogHCE *in vivo*, we performed chromatin immunoprecipitation (ChIP) on proliferating (GM) and differentiated C2C12 cells (DM) treated with the PI3K inhibitor LY, using the anti-MSY3 Ab ZONAB. ChIP on proliferating C2C12 myoblasts treated with LY showed a modest enrichment for MSY3 at the myogenin promoter compared to untreated myoblasts. In comparison, C2C12 myotubes treated with LY showed an increased enrichment when compared to their untreated counterparts (Fig. 5a). This evidence suggests that Akt inactivation results in increased levels of dephosphorylated MSY3, which in turn leads to increased MSY3 binding at the myogenin promoter region.

We also wanted to determine if Akt over-expression could rescue forced MSY3 repression of myogenin in C2C12 myoblasts and myotubes. To test this, we transfected MSY3 over-expressing C2C12 pools with a plasmid expressing the constitutively active form of Akt (myristoylated Akt) [61] and measured myogenin expression at multiple differentiation timepoints. Upon Akt expression, we observed modest levels of myogenin expression at 24 h post-differentiation, which increased to levels comparable to WT C2C12 cells at later stages of differentiation (72–96 h) (Fig. 5b). These results indicate that forced Akt expression reduces the amount of dephosphorylated MSY3 found in the nucleus during forced expression, thereby reducing its repressive activity on myogenin expression in myogenic cells.

### Identification of Ser126 as the target for Akt phosphorylation that alters MSY3 nuclear/cytoplasmic trafficking and function in myogenic cells

**Fig. 6:**
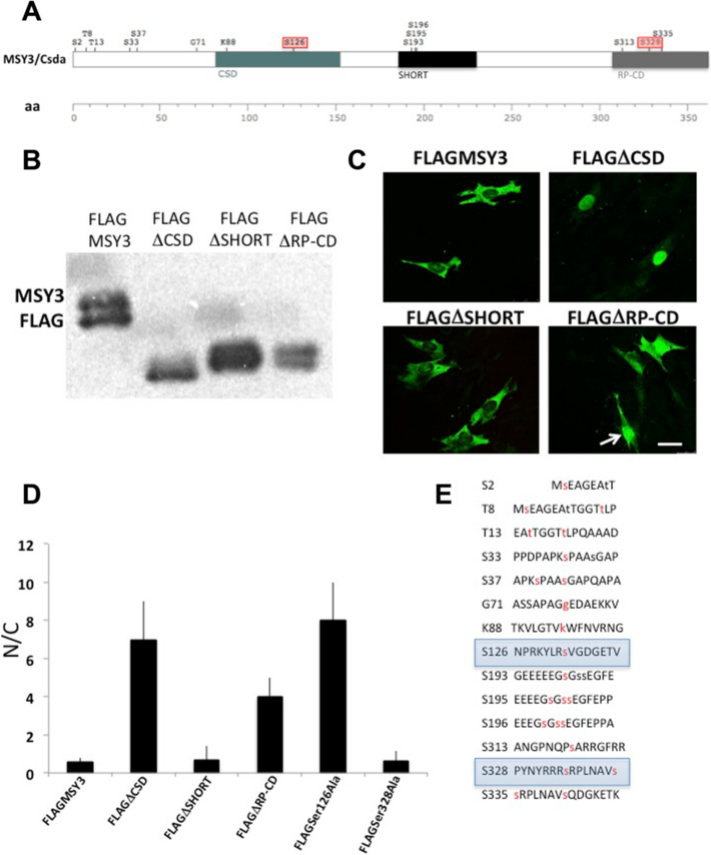
Deletion of CSD alters MSY3 nuclear/cytoplasmatic trafficking in myogenic cells. **a** Graphic illustration of the deleted MSY3 protein domains tested in the mutagenesis assay and distribution of the Akt phosphorylation consensus sites in the MSY3 protein. **b** Western blot with anti-FLAG Ab of protein extract of C2C12 cells transiently transfected with a FLAG-tagged MSY3 protein (FLAGMSY3) and FLAG-tagged MSY3 protein deleted of the cold shock (FLAGΔCSD), splicing alternative (FLAGΔSHORT), and the carboxy (FLAGΔRP-CD) domains. **c** FLAG expression tested by IF with an anti-FLAG Ab in C2C12 myoblasts transiently transfected with FLAGMSY3, FLAGΔCSD, FLAGΔSHORT, and FLAGΔRP-CD proteins. The white arrow indicates a nuclear FLAG signal of the FLAGΔRP-CD protein. Scale bar = 100 μm. **d** N/C ratios of FLAG signal in C2C12 transfected with FLAGMSY3, FLAGΔCSD, FLAGΔSHORT, FLAGΔRP-CD, FLAGSer126Ala, and FLAGSer328Ala proteins, evaluated as a mean ± DS for 60–80 cells from a total of three independent experiments. Quantification was performed as described in Methods. **e** Akt phosphorylation consensus sites in the MSY3 protein

In order to map the putative Akt phosphorylation sites, we generated FLAG-tagged WT and mutant MSY3 constructs, carrying deletions of the domains showed in Fig. 6a: CSD, alternative splicing domain (SHORT) and the carboxy (RP-CD) domain, which includes the arginine and proline-rich conserved domain and the C-terminal proline-rich domain. We introduced these constructs by transient transfection in C2C12 myoblasts and analyzed the migration of their protein products. FLAGMSY3 WT protein migrated as double bands, as expected. Treatment with AP reduced phosphorylation levels of the FLAGMSY3 WT protein similar to the endogenous MSY3 protein (Additional file 1: Figure S6). Among the mutations assayed, only the FLAG∆CSD showed a strong reduction of the upper band, indicating that the phosphorylation site responsible for MSY3 phosphorylation in C2C12 cells is located in the CSD domain (Fig. 6b). It is known that the Akt phosphorylation site in YB-1 and MSY3 in non-muscle cells was identified in the CSD. This region is highly conserved among the cold-shock protein family and is responsible for the nucleus-cytoplasmic shuttling [29, 36, 62]. In our model, we also identified the CSD as the domain responsible for the nucleus-cytoplasm trafficking. While the FLAG∆CSD protein localized exclusively in the nucleus in myoblasts, the FLAGMSY3 and the other mutated forms localized predominantly in the cytoplasm (Fig. 6c). An accurate analysis of IF (white arrow in Fig. 6c) and distribution of phosphorylated and dephosphorylated MSY3 in the nuclear (N) and (C) cytoplasmic compartments (Fig. 6d), also revealed that the deletion of the C-terminal-domain (RP-CD) induced a partial accumulation of the FLAGΔRP-CD in the nuclei. This modest nuclear migration of FLAGΔRP-CD protein suggests that the MSY3 RP-CD domain is at least partially responsible for MSY3 nucleus-cytoplasmic trafficking. For instance, it could contain another phosphorylation target, as indicated by the residual presence of the slower (phosphorylated) band in the CSD mutant (Fig. 6b).

While searching for Akt phosphorylation consensus sequences in the MSY3 protein, we did not find any site matching the minimal requirements for the Akt consensus site RXRXXS/T [63]. However, a PhosphoSitePlus scan of the MSY3 sequence [64] highlighted several low stringency putative sites that could potentially be phosphorylated by Akt (Fig. 6e). Among these we focused on two candidate amino acids, Serine126 and Serine 328, located in the CSD and RP-CD domains, respectively. MSY3-Ser126, located in the CSD, corresponds to Ser102 of the MSY3 human homolog protein YB-1. Moreover, MSY3Ser126 was shown to be phosphorylated by Akt in leukemia cell lines [29]. It has been previously demonstrated that activated Akt phosphorylates YB-1 at Ser102, affecting its nuclear translocation and function in cancer cells [36, 37].

**Fig. 7:**
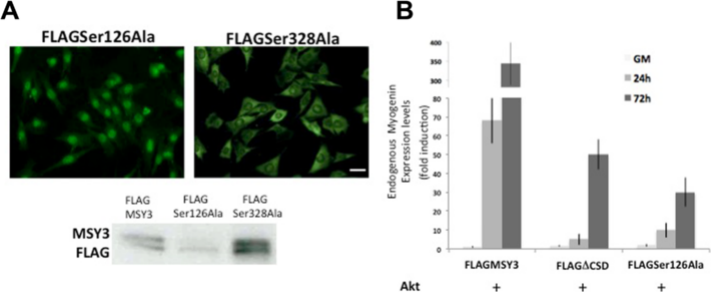
Disruption of Akt substrate Ser126 alters MSY3 nuclear/cytoplasmatic trafficking and function in myogenic cells. **a** Top: FLAG expression in C2C12 myoblasts transiently transfected with FLAG-tagged MSY3 proteins mutated at Ser126 (FLAGSer126Ala) and at Ser 328 (FLAGSer328Ala). Scale bar = 150 μm. Bottom: Western blot with anti-FLAG Ab of protein extracts of C2C12 transiently transfected with FLAG-tagged MSY3 protein (FLAGMSY3), FLAGSer126Ala and FLAGSer328Ala. **b** Measurements of myogenin expression by qRT-PCR in myrsAkt transfected C2C12 multiclones expressing FLAG MSY3, FLAGΔCSD, and FLAGSer126Ala transfected with myristoylated Akt (Akt) in proliferation medium (GM) or upon different times cultured in differentiation medium (24–72 h DM). GAPDH was used to normalize expression levels

We mutated the Serine 126 and 328 residues to Alanine in order to remove the side chain substrates and thus prevent phosphorylation by Akt. Two mutated MSY-3 constructs (MSY3 Ser126Ala) and (MSY3 Ser328Ala) were constructed and cloned in FLAG tagged vectors and were transiently expressed in C2C12 myoblasts. Nuclear accumulation of the anti-FLAG signal was observed only in myoblasts transfected with the MSY3-S126A but not myoblasts transfected with the MSY3-S328A construct. This result demonstrates that Ser126 and not Ser328 is the phosphorylation substrate responsible for the nuclear trafficking of MSY3 (Fig. 7a). Also, only Ser126A migrated as a single band, similar to the CSD mutation, providing further evidence that the substrate responsible for MSY3 phosphorylation in C2C12 is Ser126.

We wanted to further determine if Akt over-expression was able to rescue endogenous myogenin expression in the presence of the MSY3 mutants (∆CSD and Ser126A) in C2C12 myoblasts and myotubes. We transfected mock N-p3XFLAG-CMV and FLAGΔCSD and FLAGSer126Ala expressing cell pools with myristoylated Akt and measured endogenous myogenin expression levels by qRT-PCR in growth and differentiation conditions (Fig. 7b). In the myristoylated-Akt transfected cell pools expressing MSY3 mutant constructs, endogenous myogenin is strongly reduced relative to those expressing MSY3 WT. These results demonstrate that the CSD and specifically Ser126, in the CSD, mediate Akt phosphorylation of MSY3 and impair its repression of myogenin.

### Forced phosphorylation of MSY3 in skeletal muscle tissue abolishes its binding activity at the myogenin promoter

**Fig. 8:**
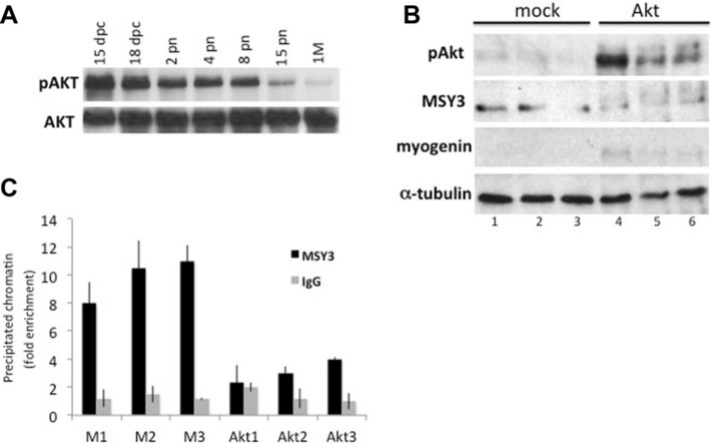
Forced phosphorylation of MSY3 in skeletal muscle tissue abolishes its binding activity at the myogenin promoter. **a** Western blot with anti-phospho and anti-total Akt of protein extracts of limb and TA muscle isolated at different developmental stages, fetal (dpc), and post-natal days (pn) and mature 1 month (1 M). **b** Western blot with anti-MSY3 Ab (ZONAB), anti-phospho-Akt, and anti-myogenin of protein extracts of TA muscle, electroporated with a mock expression plasmid pcDNA3 (mock) and with myristoylated Akt (Akt). Three replicates from three independent electroporations are shown in lanes 1, 2, 3 and lanes 5, 6, 7. Normalizer is α-tubulin. **c** Chromatin immunoprecipitation (ChIP) analysis for control IgG and MSY3 antibodies used individually to enrich fixed chromatin from TA electroporated with a mock expression plasmid (M) and with myristoylated Akt (Akt). Replicates from three independent electroporations are shown for mock plasmid electroporation (M1, M2, M3) and for myristoylated Akt (Akt1, Akt2, Akt3). qRT-PCR is used to quantify sequences from the myogenin promoter

Our next step was to determine if Akt was able to regulate MSY3 phosphorylation and activity in mature skeletal muscle, as observed in myogenic cells. To answer this question, we forced Akt expression in muscle fibers by electroporating a TA muscle of an adult mouse (3 months old, 3MM), with a myristoylated Akt construct (myrsAkt) or a control plasmid (pcDNA3, mock). Phosphorylated Akt is present at very low levels in adult muscle, since it is dephosphorylated as muscle matures (Fig. 8a). When myristoylated, Akt is over-expressed in muscle and phosphorylates MSY3, as demonstrated by the shift in the MSY3 protein migration pattern (Fig. 8b). We also detected a slight increase in myogenin expression under this condition (Fig. 8b).

Next, we determined if this forced MSY3 phosphorylation, mediated by constitutive Akt over-expression, impaired its binding at the myogenin promoter in mature muscle. To test this, we performed ChIP with ZONAB on TA muscle electroporated with myrsAkt. We observed that, indeed, this forced phosphorylation of MSY3 reduced its occupancy at the myogenin promoter in adult muscle (Fig. 8c), leading to myogenin up-regulation. These data suggest that MSY3 can be phosphorylated by Akt in adult muscle (as observed in myogenic cells *in vitro*) and as a consequence both its DNA binding activity and thereby its function as a repressor, are impaired.

## Discussion

### Dephosphorylation regulates MSY3 activity in adult muscle

In skeletal muscle, a fine-tuned regulation of myogenin expression is required. Muscle functionality depends on the correct regulation of this muscle regulatory factor, from the earlier stages of development until maturity, in normal and in pathological conditions. In Berghella et al. [16], we proposed a new regulatory pathway, which involves the Y-box protein MSY3/Csda as a negative regulator of myogenin expression in adult muscle. This study further suggested that muscle functions in postnatal life, in which myogenin plays a determinant role, are under the partial control of MSY3. The signaling pathway that influences MSY3 activity of myogenin regulation from birth to adulthood is not known [16].

During the progression from fetal to postnatal life and to late stages of muscle maturation, the phosphorylation state of the MSY3 long isoform changed, precisely during the second week after birth. Around this time, we saw a progressive reduction in myogenin expression. Therefore, the gradual MSY3 dephosphorylation correlates with a progressive down-regulation of myogenin. This concurrence strongly supports our hypothesis that MSY3 acts as a repressor when it is dephosphorylated. In the same time window, we also observed a progressive induction of both the long and short MSY3 isoforms, although in this study we did not explore the mechanisms responsible for MSY3 transcriptional regulation. The MSY3 short isoform does not have the sixth exon but retains the intact CSD domain. Unlike the long isoform, the MSY3 short isoform did not show a high rate of phosphorylation compared to the long isoform and seems to be regulated only at the transcriptional level [16]. Hence in this study, we focused only on the phosphorylation of the long isoform. However, a more accurate analysis will establish if technical reasons prevented the detection of the phosphorylated MSY3 short isoform. Phosphorylation has been described as the main post-transcriptional regulatory mechanism of the Y-box proteins. Indeed, they are targets for different signaling pathways mediated by protein kinases, which modulate or silence Y-box protein function as transcriptional repressors. In this context, ERK2/MAPK and PI3K/Akt are the most well-described pathways, the former in increasing the Y-box binding at their targets, and the latter in inhibiting it [24, 29, 35–38]. In this study, we focused on the PI3K/Akt pathway, which we demonstrated is responsible for MSY3 phosphorylation and inhibition of its activity as a repressor of myogenic gene expression and myogenic differentiation in adult muscle. We do not exclude the hypothesis that other phosphorylation pathways, also mediated by phosphatase proteins, may influence MSY3 function in different stages of skeletal muscle development. For instance, it has been recently reported that serine/threonine protein phosphatase type-1 (PP1) associates with the cold shock domain of MSY3 [65].

### Akt regulates myogenin expression and myogenesis by MSY3 phosphorylation

In the present work, we discovered a signaling mechanism linking the PI3K/Akt pathway to regulation of myogenin expression through phosphorylation of MSY3. Analysis of the MSY3 protein migration pattern by Western blot, from differentiating C2C12 myogenic cells, showed an increase in the phosphorylated MSY3 long isoform. This shift was coincidental with the myogenin expression increase and completion of myogenic differentiation. From our previous studies, we know that MSY3 binds the myogHCE in the myogenin promoter and represses myogenin expression in only proliferating and undifferentiated C2C12 myoblasts and not in myotubes. Moreover, knocking-down MSY3 expression in myoblasts induced myogenin expression [16]. Our present study suggests that in order to allow myogenin expression, MSY3 needs to be inactive and its phosphorylation can be one of the mechanisms by which its function is inhibited in differentiated muscle. Also, MSY3 is strongly expressed in the limbs of developing embryos [66]. At this stage MSY3 is phosphorylated and myogenin is highly expressed in somites and in limbs. Based on these data, we postulate that when MSY3 is phosphorylated, it is inactive as a repressor and is predominantly sequestered in the cytoplasm, thus allowing myogenin expression.

Multiple reports suggest that Y-box proteins are regulated through Akt/PKB-mediated phosphorylation of the cold shock domain, which is responsible for their regulatory activity at the transcription and translation level. Phosphorylation by Akt, indeed, impairs the inhibitory activity of the MSY3 homolog, MSY1 (YB-1), and of MSY3 (YB-2) on oncogenesis progression [29, 36–38]. So, we asked if Akt could also control MSY3 phosphorylation in skeletal muscle. We showed that treatment of C2C12 cells with the P13K/Akt specific inhibitor, LY294002 (LY), reduced the phosphorylated MSY3 fraction, both in proliferating and differentiated myogenic cells. siRNA-mediated depletion of Akt has the same effect, suggesting a role for Akt in modulating MSY3 activity during muscle differentiation. In a cell culture myogenic system, we demonstrated that silencing Akt reduced phosphorylated MSY3 levels and concomitantly myogenin expression and muscle differentiation. We showed, using a kinase assay, that Akt directly and specifically phosphorylates recombinant MSY3 *in vitro*. While MSY3 might be a GSK3β target since it contains potential GSK3β sites in the CSD, in our assay it is not phosphorylated by recombinant GSK3β, in agreement with previous results [24]. Upon phosphorylation by Akt, MSY3’s ability to bind its consensus binding site (myogHCE) on the myogenin promoter *in vitro* is impaired, suggesting a partial role for Akt phosphorylation in destabilizing the MSY3-myogenin promoter binding complex formation. Moreover, when Akt activity is blocked by treatment with LY in differentiating C2C12 cells, MSY3 is able to bind myogHCE, and represses myogenin expression, but not in untreated C2C12 cells. Additionally, over-expression of the myristoylated form of Akt restored myogenin expression in MSY3 over-expressing myotubes. Altogether, these results demonstrate that MSY3 phosphorylation by Akt ensures correct progression of muscle differentiation, by regulating myogenin expression. When MSY3 is phosphorylated, its affinity for myogHCE is reduced and it is sequestered in the cytoplasm. Cytoplasmic sequestration then allows the transcription regulatory factors Pbx and MyoD to access the MSY3 consensus site, thus promoting chromatin opening and gene activation, according to the model proposed previously [16, 67].

A role of Y-box protein in myogenic differentiation has also been described previously for YB-1 (MSY1), as a negative regulator of MyoD expression by binding to the MyoD core enhancer, cooperatively with a transcriptional repressor, Msx-1 [68]. Moreover, initial evidence that MyoD might also be directly affected by MSY3 was previously reported, indicating that both MyoD and myogenin are modulated by the same repressor complex [16]. We consider the appealing possibility that Akt could regulate the nuclear localization of a group of Y-box proteins, which in turn target myogenic genes, sequestering them in the cytoplasm thus promoting muscle differentiation.

Several studies have explained how the PI3K/Akt pathway affects muscle differentiation through different mechanisms: by phosphorylation and activation of transcription factors (MyoD) and cofactors (MEF2) or of different components of the muscle transcriptosome (acetyltransferases, p300, and pCAF), or of proteins that regulate terminal differentiation and myotube hypertrophy [47, 55]. In this study, we propose that the Akt/MSY3/myogenin signaling cascade acts as a promyogenic negative regulatory circuit that proceeds in parallel with the other positive regulatory pathways controlled by Akt during muscle differentiation. Hence based on our findings, PI3K/Akt signaling not only promotes myogenesis but also actively avoids repression of muscle differentiation. Previous studies have shown that Akt-mediated phosphorylation in other contexts can directly or indirectly block the activity of negative regulators of transcription such as C/EBPα or the Polycomb group protein Bmi1 to promote cell proliferation or tumor growth [69, 70]. Moreover, PI3K/Akt pathway inactivation results in the conversion of chromatin from permissive to repressive, as demonstrated by association of the Polycomb-associated methyltransferase Ezh2 or in an association of MEF2 with myogenesis inhibitors (Ezh2 and HDAC4) instead of myogenesis activators [55]. Our data suggests a new mechanism of action by which the PI3K/Akt pathway prevents myogenic differentiation. Ultimately, Akt acts by direct phosphorylation and inactivation of a class of negative regulators of muscle differentiation, the Y-box proteins.

### Akt phosphorylation regulates MSY3 nuclei/cytosol translocation

The underlying mechanism of action mediated by Akt phosphorylation to exert its function often affects the substrate sub-cellular trafficking [34, 54]. In cases such as FOXO, Akt phosphorylation dictates exclusion of the phosphorylated transcription factors from the nuclei, leading to inhibition of their transcriptional regulatory activity [32]. Altering sub-cellular localization is also the manner in which Akt-mediated phosphorylation modulates Y-box protein functions. Several lines of evidence show that Y-box proteins act as transcriptional regulators of genes involved in proliferation and differentiation, when they translocate to the nucleus and as regulators of translation or different functions, when they are in the cytoplasm [36, 37, 62, 71]. We observed that inhibiting Akt activity by LY treatment, deleting the CSD, and mutating the Akt phosphorylation site at Ser126 in the CSD induced MSY3 protein migration from the cytoplasm to the C2C12 myogenic cell nucleus. Although both Ser126 and Ser328 residues are low stringency predicted Akt targets compared to the Akt consensus site (RXRXXS/T), the Ser126 site is conserved among vertebrates from human to zebrafish. This region also falls into the highly conserved CSD domain of the Y-box protein, which is responsible for binding DNA. This could explain why among the two putative sites, Ser126 is the substrate for Akt phosphorylation in MSY3, thus responsible for its nuclear to cytoplasmic migration. Deletion of the C-domain (ΔRP-CD) induced a partial migration of flagged MSY3 protein in the nuclei, but was confirmed not to be due to the ablation of the putative Akt phosphorylation site Ser328. It is possible that the partial migration might have been a result of the deletion of a region within RP-CD, responsible for intracellular trafficking or for the conformation and stability of the protein. Nevertheless, we did not analyze other Akt putative target sites located in the RP-CD that could account for the partial nuclear migration observed. Although, we identified the Akt phosphorylation substrate in MSY3 partially responsible for its nuclear-cytoplasm translocation, we have yet to determine the mechanism and the players involved in its subsequent subcellular trafficking. As muscle fibers mature, MSY3 shifts from the phosphorylated to the dephosphorylated form and translocates from the cytosol to the nuclei, and coincidently, phosphorylated active Akt is reduced. These results lead us to believe that the same mechanism of Akt-mediated phosphorylation of MSY3, determined in myogenic cell culture, is active during muscle development. We also postulate that the MSY3 nuclear-cytoplasmic shuttling observed in postnatal muscle is correlated to its function in repressing myogenin and other possible targets involved in muscle maturation and growth.

### Function of Akt/MSY3 regulatory circuit in adult muscle

The most well-documented role of Akt in skeletal muscle is to induce hypertrophy in muscle fibers and maintain muscle mass during development, by controlling protein synthesis in muscle fibers, by promoting proliferation and activation of muscle satellite cells, and by counteracting atrophy [39, 40]. The negative control exerted by Akt on MSY3 function as a repressor could be crucial during perinatal and early postnatal life, when skeletal muscle grows dramatically and concomitantly with increased body mass. Contrarily, upon maturity, when skeletal muscle achieves the right size and it needs to be maintained, Akt-mediated phosphorylation of MSY3 is reversed and it is employed in silencing its target genes. Myogenin is down-regulated in adult and unperturbed myofibers. When we forced Akt expression in adult muscle fibers, we observed phosphorylation of MSY3 protein with a subsequent over-expression of myogenin. The phosphorylated MSY3 showed reduced occupancy at the myogenin promoter thereby allowing myogenin reactivation. Forced Akt expression was confined to muscle fibers and not observed in satellite cells, and it was accompanied by an increase of muscle fiber size (data not shown) as already described [44, 72]. This evidence suggests that the Akt/MSY3 regulatory axis may be involved in a possible mechanism to control muscle size within myofibers. It is possible that upon phosphorylation, MSY3 binding of other genomic targets required for skeletal muscle growth and maturation is also impaired. This is plausible considering that myogenin deletion in early postnatal life does not affect skeletal muscle growth [4], and Akt/MSY3 might control mechanisms independent of myogenin in this context. Further genome-wide studies on MSY3 occupancy will address this point.

In our previous work, we proposed a role of MSY3/Csda in neuromuscular junction (NMJ) development occurring during skeletal muscle innervation due to its inhibitory action on myogenin, which positively activates AChRs along muscle fibers [16]. Other mechanisms have been described that respond to nerve signals in order to down-regulate myogenin and involve transcriptional repressors [12, 13, 31]. As Akt regulates MSY3 function, it is likely that the PI3K/Akt pathway participates in nerve-mediated signaling, occurring during motor innervation. Nevertheless, a link between nerve activity and Akt activation has not been described yet. It has been well described how Akt activation can be promoted by intracellular calcium increase [73, 74], although the signaling cascade that precedes the dephosphorylation of MSY3 in mature muscle and whether other MRFs are involved in this timed process is currently unknown. Additionally, it is possible that other post-translational modifications of the MSY3 protein could act in order to connect electrical activity of the nerve to myogenin via MSY3.

In adult life, myogenin is reinduced following injuries in satellite cells or upon resection of the sciatic nerve (denervation) in muscle fibers [57, 75]. The PI3K signaling pathway plays an active role in response to regeneration of damaged muscle. In satellite cells, Akt promotes muscle gene expression and terminal differentiation through different cooperating targets [55, 76, 77]. It is plausible that the Akt/MSY3/myogenin regulatory circuit drives myogenesis in satellite cells upon injury and thus contributes to muscle fiber growth through mechanisms mediated by satellite cells.

Some studies showed that when denervation is induced in muscle, active pAkt and total Akt are significantly and unexpectedly up-regulated [78]. The reason for Akt activation still remains unknown, although it can be explained as a compensatory effect to counteract denervation-induced muscle atrophy. Indeed, constitutively active Akt can inhibit neurogenic atrophy [43]. Additionally, we have previously shown that MSY3 protein levels start to decrease upon denervation followed by an increase in myogenin and Myod expression levels. Conversely, it was shown that forced expression of MSY3 (electroporated) in denervated muscle led to a knockdown of myogenin, Myod, and α AChR expressions with no effect on MCK and ℇ AChR expressions [16]. When reactivated, Akt could phosphorylate MSY3 and hence promote myogenin or other MSY3 targets derepression upon nerve section. In this study, we did not explore the downstream effects of Akt over-expression in muscle fibers, although using transgenic models over-expressing Akt will help elucidate the role of the Akt/MSY3 regulatory circuit in the conditions when myogenin is re-expressed.

## Conclusions

Altogether, these results propose a new mechanism of action for the PI3K/Akt pathway in controlling myogenic differentiation and possibly adult muscle function by direct phosphorylation of MSY3. In a myogenic cell culture model, we demonstrated that Akt modulates the activity of MSY3, a repressor of myogenic differentiation. Akt phosphorylation of MSY3 inhibits its repression of myogenin, thereby turning on myogenin expression upon differentiation. In adult myofibers, the Akt/MSY3 regulatory circuit is involved in down-regulating and maintaining low myogenin expression levels, in turn controlling adult skeletal muscle growth and metabolism. These results support the hypothesis that the key components of the PI3K/Akt signaling pathway that control myogenin expression in muscle fibers are potential therapeutic targets for therapies to control muscle regeneration and degeneration.

## Additional file

Additional file 1:
**Supplementary figures.** **Figure S1.** WB with anti-myogenin Ab (FD5) of nuclear fraction protein extracts of limbs and TA isolated at embryonic, fetal (dpc), postnatal (pn) days and mature (1M) stages. Normalizer is Histone H3. **Figure S2.** A) WB with anti-MSY3 Ab (ZONAB) of immunoprecipitated MSY3 (IP-MSY3) from protein extracts of 8 pn and 15 pn limbs. B) WB with ZONAB of IP-MSY3 from 8 pn limb protein extracts treated with AP. **Figure S3.** MSY3 and caveolin 3 IF and Hoechst staining on cross sections of limb of 2 pn pups and 1M mice TA. Merged images are shown in Fig. 1d. Scale bar 2 pn = 100 μm. Scale bar 1M = 200 μm. **Figure S4.** A) MHC IF and Hoechst staining of C2C12 (48h DM) treated with LY (20μM), wortmannin (50μM), rapamycin (50μM), and SB (10μM). (Scale bar = 400 μm). B) WB with ZONAB of C2C12 treated with LY and SB. C) WB with ZONAB of C2C12 treated with LY, wortmannin (W) and rapamycin (R). D) WB with ZONAB of C2C12 treated with KN-62 and KN-93. E) WB with ZONAB of C2C12 treated with PD98059 (PD). Normalizer is α-tubulin. F) IF of MHC, and Hoechst staining of C2C12 (36h DM), transfected with Akti oligos, Scale bar = 400 μm. **Figure S5.** A) ZONAB and FD5 IF and Hoechst staining of C2C12 cells, LY untreated (−) and treated in GM and DM (48h). Scale bar = 200 μm. For ZONAB IF, a 40X magnification is also shown. *White arrow* indicates sporadic selective nuclear signal in LY untreated cells; *green arrow* indicates cytoplasmic-nucleus shuttling in myotubes upon LY treatment. Scale bar = 100 μm. B) WB of nuclear (N) and cytosolic (C) fraction of LY treated C2C12. Normalizers are histone H3 (N) and GAPDH (C). **Figure S6.** WB with anti-FLAG Ab of a C2C12 multiclone expressing FLAG-tagged MSY3 protein extracts treated with AP.

## Abbreviations

AChR: acetylcholine receptor
AP: Antarctic Phosphatase
BSA: bovine serum albumin
C/EBPα: CCAAT-enhancer-binding protein
CAMKII: calcium/calmodulin-dependent protein kinase
Csda: cold shock domain A
DM: differentiation medium
ECL: electrochemiluminescence
EMSA: electrophoretic mobility shift assay
ERK2: extracellulars signal-regulated kinase
Ezh2: histone-lysine N-methyltransferase
FBS: fetal bovine serum
FoxO3a: forkhead box O3
GM: growth medium
GSK-3β: glycogen synthase kinase 3β
GST: glutathione-s-transferases
HDAC: histone deacetylases
HS: horse serum
IF: immunofluorescence
IGF: insulin-like growth factor
IP: immunoprecipitation
MAPK: mitogen-activated protein kinase
MEF2: myocyte enhancer factor 2
MEK: mitogen-activated protein kinase kinase
MHC: myosin heavy chain
MRFs: myogenic regulatory factors
mTOR: mammalian target of rapamycin
MyoHCE: myogenin highly conserved element
NGF: normal goat serum
NMJ: neuromuscular junction
PBS: phosphate-buffered saline
pCAF: P300/CBP-associated factor
PI3K: phosphatidylinositol 3-kinase
PP1: serine/threonine protein phosphatase type-1
qRT-PCR: quantitative real-time polymerase chain reaction
SDS-PAGE: sodium dodecyl sulfate-polyacrylamide gel electrophoresis
TA: tibialis anterior
VEGF: vascular endothelial growth factor
WB: Western Blot
