# Supplementary figures and images for: Akt-mediated phosphorylation controls the activity of the Y-box protein MSY3 in skeletal muscle
Source: Skelet Muscle. 2015 May 29;5:18. doi: 10.1186/s13395-015-0043-9 (PMC4491233; doi:10.1186/s13395-015-0043-9)

## Histone H3

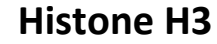

Fig S2

A

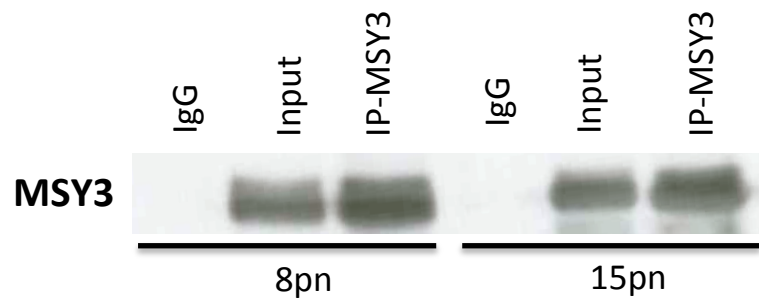

B

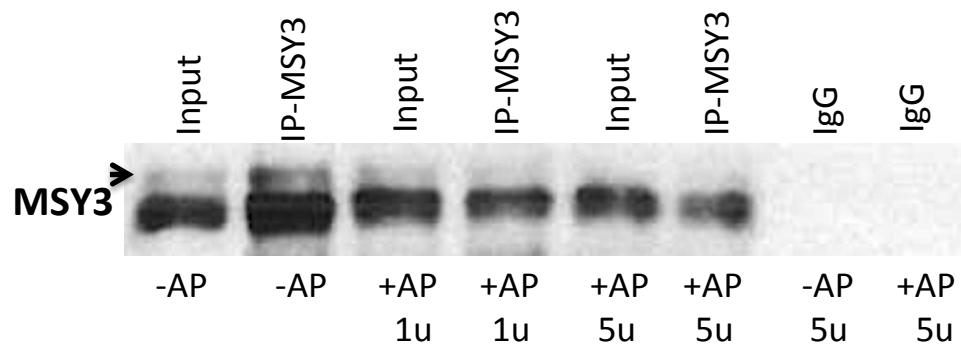

Fig S3

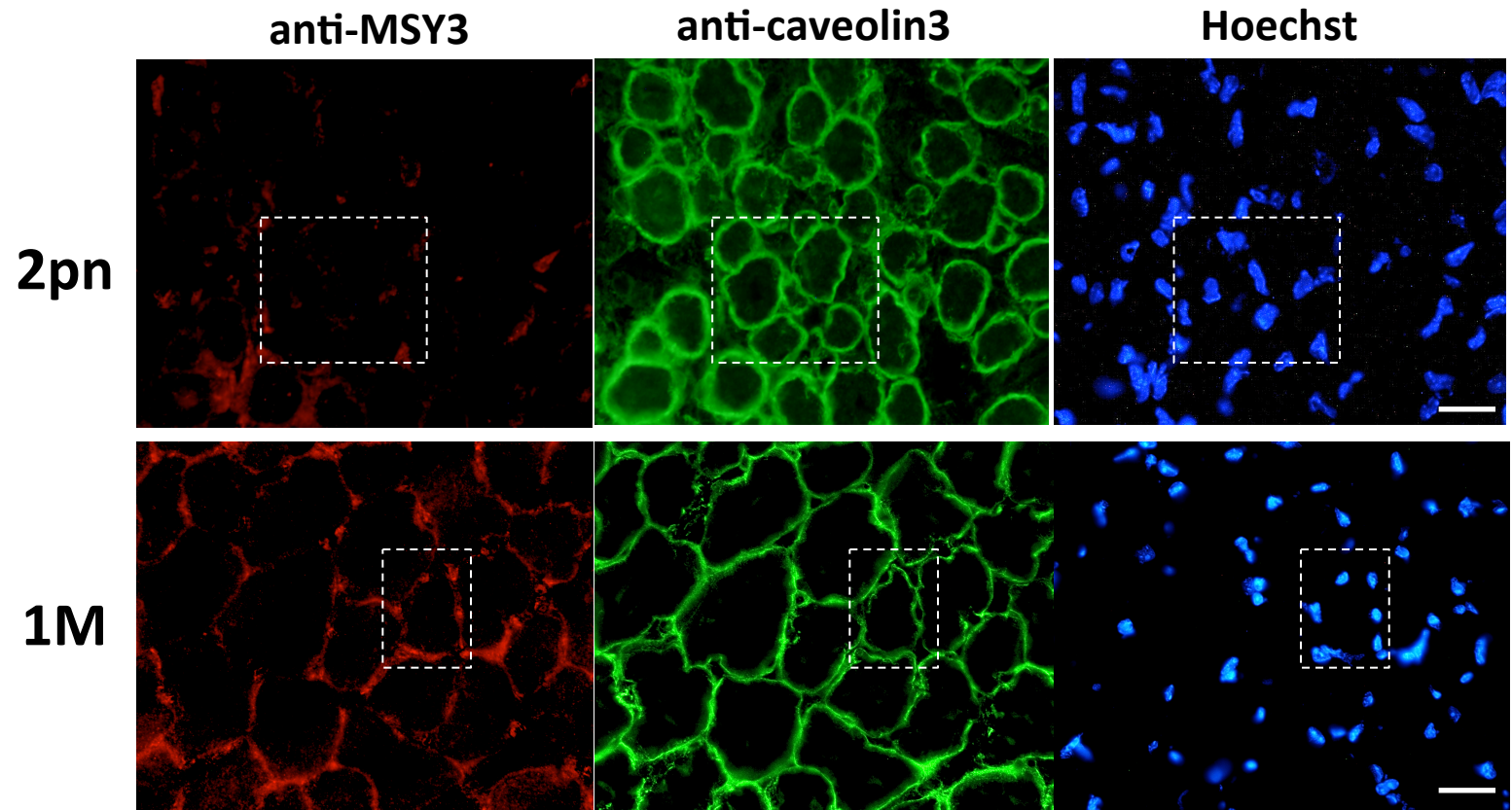

Fig S4

A

- LY29002 wortmannin rapamycin SB203580

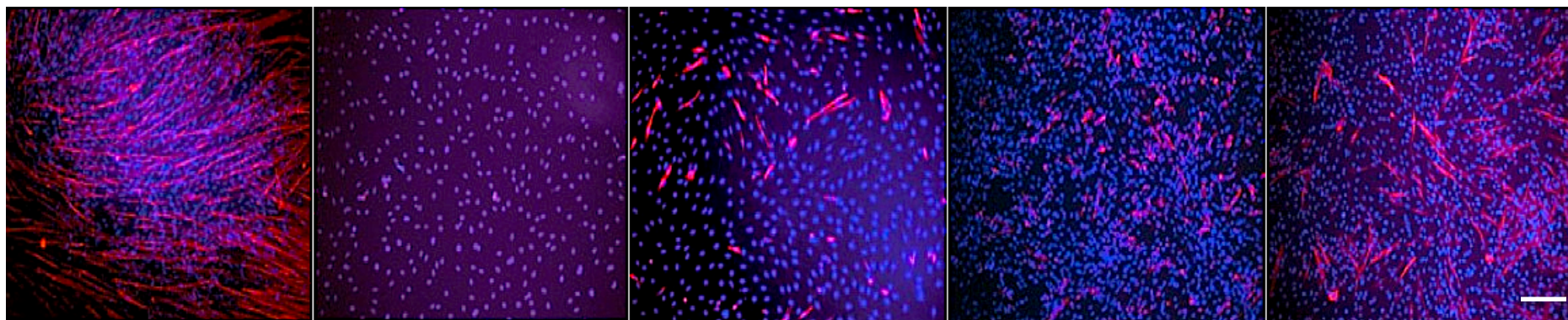

B

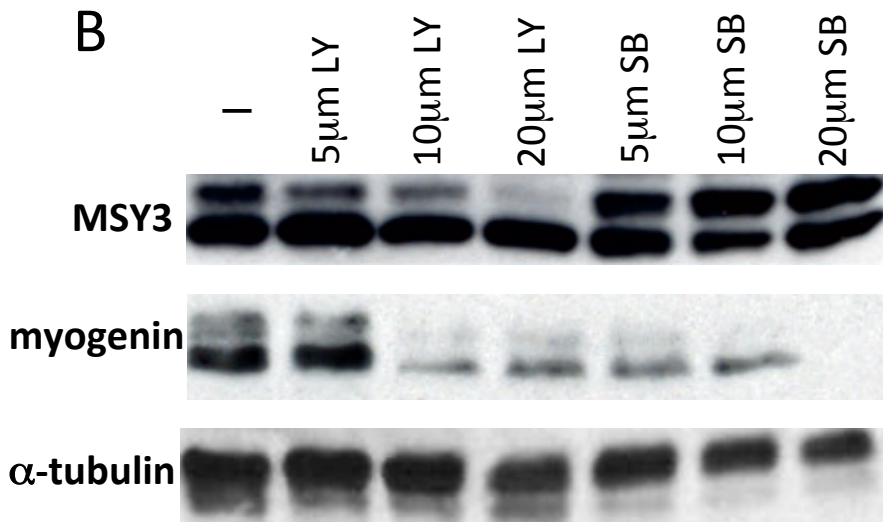

C

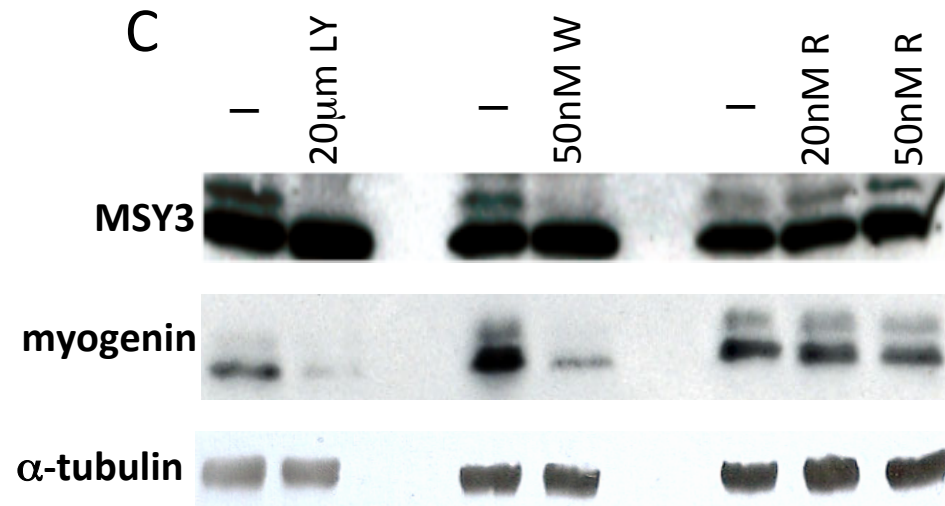

D

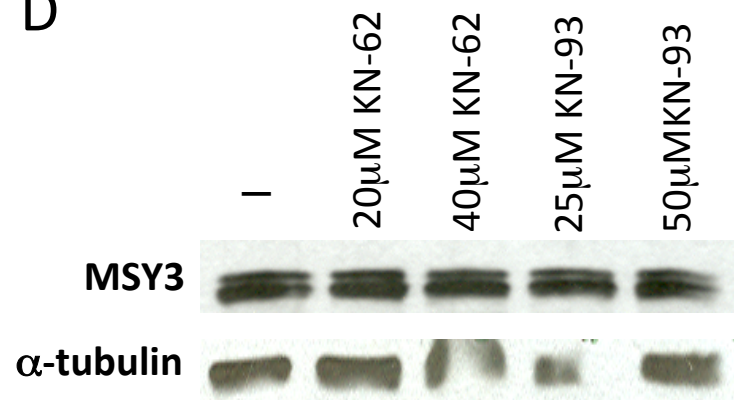

E

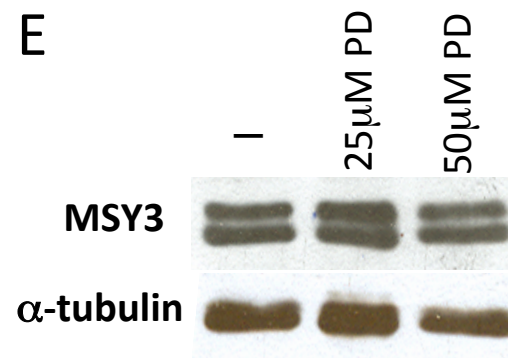

F

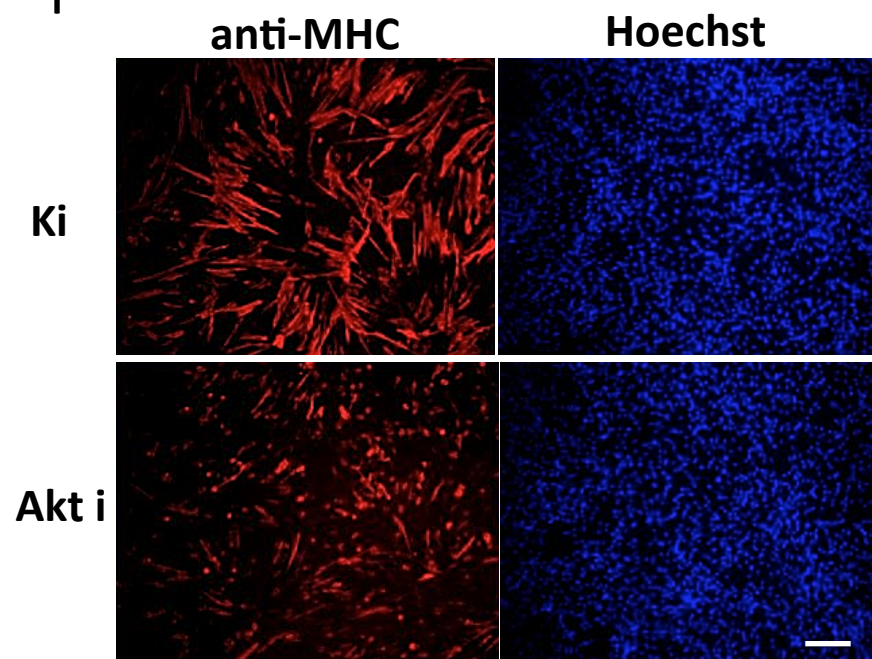

A

**GM**

## Hoechst

---

LY

**DM**

---

**LY**

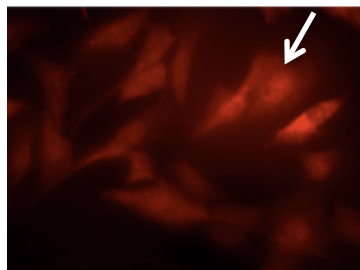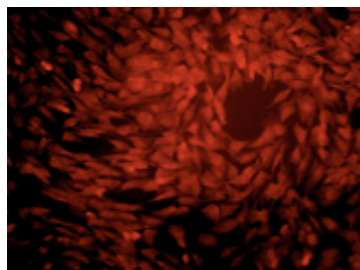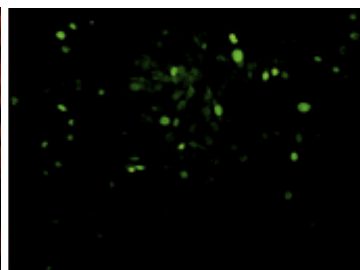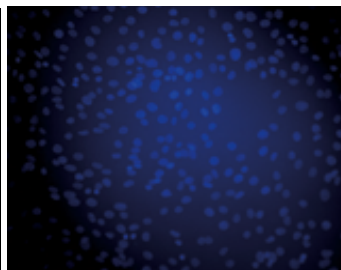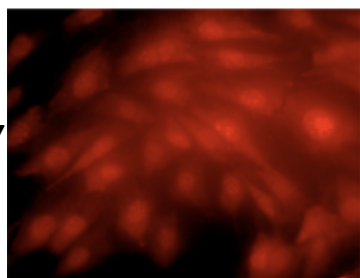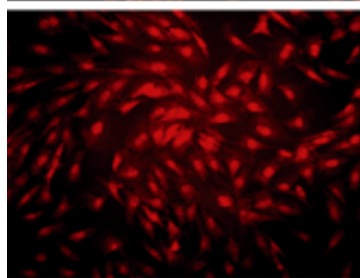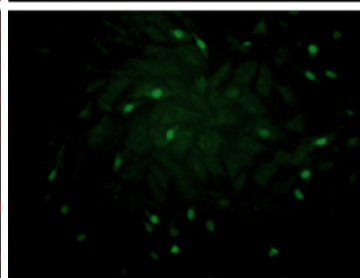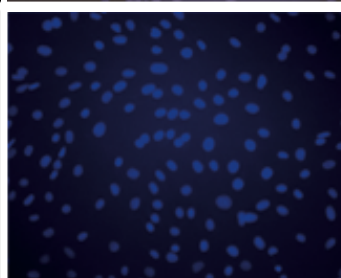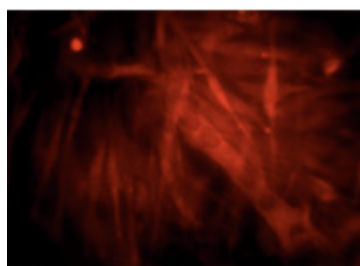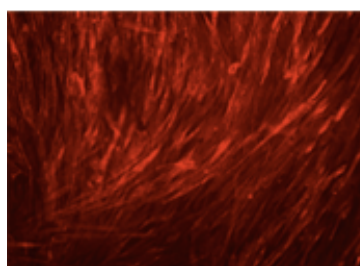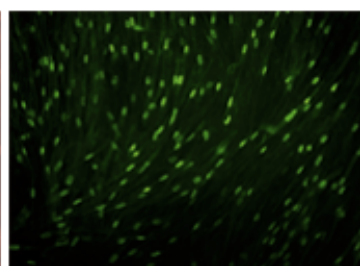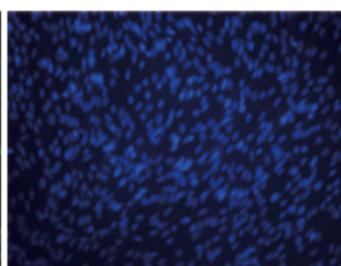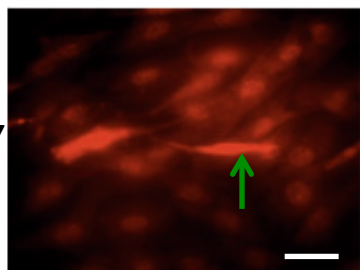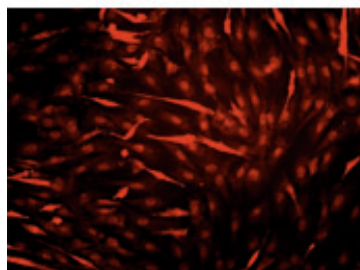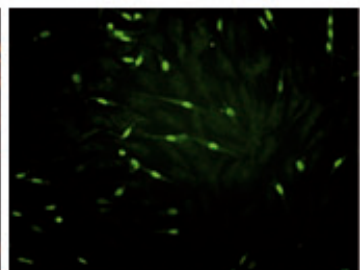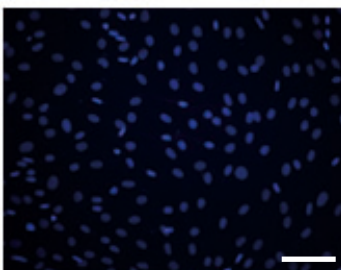

**B**

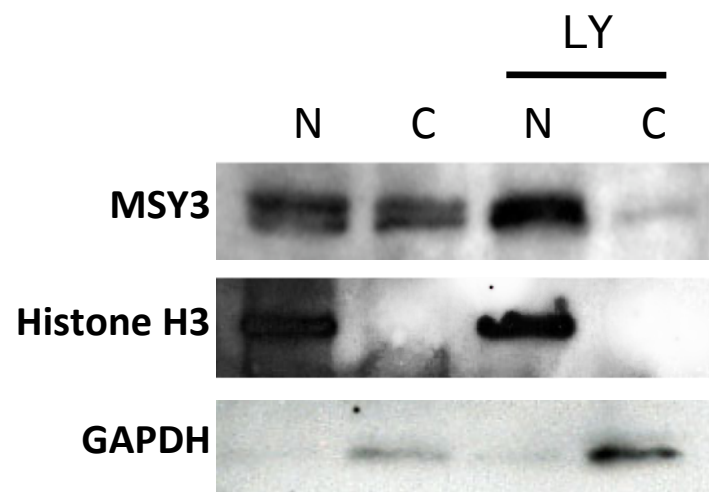

Fig S6

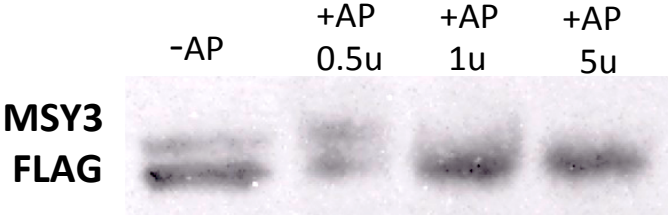

Supplement: Additional file 1: — Supplementary figures. Figure S1. WB with anti-myogenin Ab (FD5) of nuclear fraction protein extracts of limbs and TA isolated at embryonic, fetal (dpc), postnatal (pn) days and mature (1M) stages. Normalizer is Histone H3. Figure S2. A) WB with anti-MSY3 Ab (ZONAB) of immunoprecipitated MSY3 (IP-MSY3) from protein extracts of 8 pn and 15 pn limbs. B) WB with ZONAB of IP-MSY3 from 8 pn limb protein extracts treated with AP. Figure S3. MSY3 and caveolin 3 IF and Hoechst staining on cross sections of limb of 2 pn pups and 1M mice TA. Merged images are shown in Fig. 1d. Scale bar 2 pn = 100 μm. Scale bar 1M = 200 μm. Figure S4. A) MHC IF and Hoechst staining of C2C12 (48h DM) treated with LY (20μM), wortmannin (50μM), rapamycin (50μM), and SB (10μM). (Scale bar = 400 μm). B) WB with ZONAB of C2C12 treated with LY and SB. C) WB with ZONAB of C2C12 treated with LY, wortmannin (W) and rapamycin (R). D) WB with ZONAB of C2C12 treated with KN-62 and KN-93. E) WB with ZONAB of C2C12 treated with PD98059 (PD). Normalizer is α-tubulin. F) IF of MHC, and Hoechst staining of C2C12 (36h DM), transfected with Akti oligos, Scale bar = 400 μm. Figure S5. A) ZONAB and FD5 IF and Hoechst staining of C2C12 cells, LY untreated (−) and treated in GM and DM (48h). Scale bar = 200 μm. For ZONAB IF, a 40X magnification is also shown. White arrow indicates sporadic selective nuclear signal in LY untreated cells; green arrow indicates cytoplasmic-nucleus shuttling in myotubes upon LY treatment. Scale bar = 100 μm. B) WB of nuclear (N) and cytosolic (C) fraction of LY treated C2C12. Normalizers are histone H3 (N) and GAPDH (C). Figure S6. WB with anti-FLAG Ab of a C2C12 multiclone expressing FLAG-tagged MSY3 protein extracts treated with AP. [file 13395_2015_43_MOESM1_ESM.pdf]
